# Assessment of patient safety culture among paramedical personnel at general and district hospitals, Fayoum Governorate, Egypt

**DOI:** 10.1186/s42506-019-0031-8

**Published:** 2020-01-31

**Authors:** Naglaa A. El-Sherbiny, Eman H. Ibrahim, Wafaa Y. Abdel-Wahed

**Affiliations:** Public Health Department, Faculty of Medicine, Fayoum University, Gamma St., Keman Square, Fayoum, Egypt

**Keywords:** Patient safety, Barriers, Paramedical, Fayoum Governorate

## Abstract

**Background:**

Healthcare is a high-risk industry that requires regular assessment of patient safety climate within healthcare organizations. This addresses the organizational cultural issues and explores the association between organizational climate and patient outcomes. This study aimed to assess patient safety culture among paramedical health employees at Fayoum general and district hospitals and to determine factors affecting their perception of patient safety.

**Methods:**

A descriptive cross-sectional study was conducted at the general hospital and four district hospitals in Fayoum Governorate, Egypt, among 479 paramedical healthcare workers. The standardized Hospital Patient Safety scale (HSOPSC) that composed of 12 safety culture dimensions was used.

**Results:**

The mean total safety score varies according to the participant’s position and work area. The total patient safety score was 46.56%. No dimension reported score above 75%. The highest mean composite scores were for organizational learning and continuous improvement (65.36%) and teamwork within hospital units (63.09%). The lowest reported score was for communication openness (17.9%). More perception of safety dimensions was seen in females than males, participants in direct contact with patients, and those with work experience less than 10 years.

**Conclusion and recommendations:**

Overall, the degree of patient safety is low at Fayoum public hospitals. No dimension scored above 75%, and 7 out of 12 dimensions scored less than 50%. Hence, continuous monitoring and updating of the ways of incident reporting is highly recommended. This may be done through setting up a web-based incident reporting system accessible for 24 h.

## Introduction

The concept in patient safety (PS) is to reduce the risk and completely avoid the preventable one that is associated with provided healthcare [1]. The European Society for Quality in Health Care defined the dynamic culture of PS as an integrated pattern of individual and organizational behavior, based upon shared beliefs and values that continuously seek to minimize the patient harm, which may result from the process of care delivery [2]. Prevention of harm could be achieved through preventing errors and learning from these errors with emphasis on involving the healthcare professionals, organizations, and patients [3].

According to the OECD (2018) report, the situation in low- and/or middle-income countries (LMICs) is more critical, as about 2.6 million deaths happen as a result of 134 million adverse events occur in hospitals annually [4]. PS requires knowledge and skills in multiple areas, including human factors and systems management as most common errors, which are preventable, are related to these areas including investigation errors, medication errors, and nosocomial infections [5].

The safety culture of an organization is the outcome of individual and organization’s values, attitudes, perceptions, competencies, and patterns of behavior that determine the commitment to, and the style and proficiency of, an organization’s health and safety management. Therefore, full understanding and targeting of attitudes and behaviors related to PS are required to support a culture of safety and obtain a desirable outcome in all organizational aspects [6]. All these could be achieved through setting up clear policies, having skilled healthcare professionals, all-level leadership, up-to-date data, and patient-centered care in order to maintain healthcare safety sustainability [1]. Assessing the existing safety culture allows organizations to obtain a clear view of patient safety aspects requiring urgent attention, identify the strengths and weaknesses of their safety culture, and enhance continuous quality management [7]. In most Arab countries, PS is considered a major issue for health policy makers, which necessitates identifying and analyzing its negatively contributed factors [8].

In Egypt, the nurse/patient ratio is half the international figure, which led to a significant shortage in qualified staff who may not have a clear view of safety framework. The potentiality for safety hazards can increase if they work in a challenging and dynamic environment with high workload. Additionally, the paramedics were available and easily approachable for data collectors [9]. The current study is considered the first to be conducted in Fayoum Governorate to provide insight into PS culture among paramedical staff. It aimed to assess patient safety culture among paramedical health staff at general and district hospitals and determine factors affecting perception of PS.

## Subjects and methods

### Study design and setting

A cross-sectional descriptive study was carried out at Fayoum General Hospital, and four district hospitals in Etsa, Abshaway Tamia, and Senoris in Fayoum Governorate with a total number of five hospitals were included in this study. Fayoum is one of 27 Governors in Egypt, located in the southwest of Cairo with an area of 1827 km^2^ and an estimated population of 3.359.399 million [10]. The survey was conducted over a period of 4 months between May 2018 and September 2018.

### Study participants

The study population was randomly selected from five secondary-level-care hospitals run by the Ministry of Health at Fayoum Governorate/Egypt. The paramedical staff (nurses, pharmacists, and technicians) working in the five hospitals who had contact with patients, working full-time, and agreed to participate were included. Participants with < 1-month experience and part-time workers were excluded.

A total sample of 479 paramedical staff was selected. The sample size was calculated by Epi Info 2000, using the following assumption: safety level 50%, precision level 5%, and confidence interval 95%; 385 was calculated, which was increased by 20% to overcome non-response. A purposive sample technique was used, and 100 persons were selected from each hospital. The sample recruitment was carried out from both internal medicine and general surgery departments. The study obtained a high response rate of 95.8%.

### Data collection

#### Study tool

The Hospital Patient Safety scale (HSOPSC), developed by the Agency for Healthcare Research and Quality (AHRQ), was used and included 12 safety culture dimensions: 10 patient safety culture dimensions (included seven units-level dimensions and three hospital-level dimensions) and 2 outcome dimensions. The total number of items was 42, and each item was scored on a 5-point Likert scale ranging from 1 = “strongly disagree” to 5 = “strongly agree.”

The questionnaire covers two sections: demographic characteristics which included age, gender, position, years of work experience, and work area. The other section was patient culture dimensions that included teamwork within units (4 items), supervisor/manager expectations and actions promoting PS (4 items), organizational learning-continuous improvement (3 items), management support for patient safety (3 items), overall perceptions of PS (4 items), teamwork across units (4 items), staffing (4 items), handoffs and transitions (4 items), non-punitive response to errors (3 items), feedback and communication about error (3 items), communication openness (3 items), and frequency of events reported (3 items). An Arabic version of the HSOPSC questionnaire, which was submitted by Najjar et al. [11], was used. It showed that the HSOPSC is a valid and reliable instrument for assessing the safety culture in the Arabic speaking hospital settings. Furthermore, it was field-tested on a pilot sample of 60 participants (10% of the target sample). The results of the pilot sample were not included.

Data was collected by the authors via a one-on-one interview using the Arabic version of The Hospital Patient Safety scale over a period of 4 months from May to September 2018.

#### Data analysis

The domain scores were calculated by dividing the total number of positive responses in each domain by the total number of items in that domain. These scores expressed how positively people answered the items in each safety culture dimension: if the composite score for all items in the same PS dimension was more than 75%, this dimension was considered as an “area of strength”; if it was between 75 and 50%, this PS dimension was considered as “an area with potential for improvement”; and if less than 50%, considered an area of weakness [12]. The patient safety grade was estimated from respondents’ overall grading of their work area or unit as excellent, very good, acceptable, poor, or failing. Statistical analysis was performed using SPSS (Statistical Package for Social Sciences) version 21 (IBM Corp. Released 2012. IBM SPSS Statistics for Windows, version 21.0. Armonk, NY: IBM Corp). Variables were presented using numbers and percent for qualitative variables; and mean and standard deviation for quantitative variables. A comparison of the mean scores of safety scales was done using Student *t* test for two-group comparisons, and ANOVA test for more than two groups. Correlation between safety scales was performed using Pearson correlation. *p* value ≤ 0.05 was considered statistically significant.

## Results

The study participants were 479 paramedical staff; they were almost equally distributed across age groups between 25 and 54 years old as well as among gender. Two thirds of the participants (64.3%) were nurses, while the other one third were pharmacists (12.3%) and technicians (23.4%). About 52.4% of participants work mainly in outpatient services, and nearly half of them (44%) had an experience of 6–10 years of work. About 70% (69.9%) had direct contact with patients. The number of reported events ranged from none to 20; only one to two events were reported by 44.3% of participants and three to four events by 32.2% in the last year (Table 1).

**Table 1:** Basic characteristics of paramedical participants at Fayoum General Hospital and other selected district hospitals, 2018 (*n* = 479)

Category	Number	Percentage
Age in years		
< 25	55	11.5
25–34	114	23.8
35–44	113	23.6
45–54	139	29.0
≥ 55	58	21.1
Gender		
Male	237	49.5
Female	242	50.5
Job title		
Nurse	308	64.3
Technician	112	23.4
Pharmacist	59	12.3
Workplace		
Outpatients	251	52.4
Inpatients	62	12.9
Others	166	34.7
Years of work experience		
1–5 years	67	14.0
6–10 years	214	44.7
11–15 years	83	17.3
> 15 years	115	24.0
Direct contact with the patient		
Yes	335	69.9
No	144	30.1
Number of reported events in the last 12 months		
No reports	58	12.1
1–2 events	212	44.3
3–5 events reported	154	32.2
6–10 events reported	49	10.2
11–20 events reported	6	1.2

Table 2 summarizes the composite scores on 12 dimensions of patient safety culture. No dimension reported score above 75. The highest mean composite score was 65.36% for organizational learning-continuous improvement. The next highest scoring dimension was teamwork within hospital units (63.09%). Other reported dimensions above 50% were staffing work conditions (57.6%), supervisor/manager expectations and actions promoting safety (59.8%), management support for patient safety (59.5%), and handoffs and transitions (55.1%). All other safety culture dimensions had scores less than 50%. The lowest reported score was communication openness, 17.9%. The total patient safety score was 46.56%. The mean total safety score varies according to the participants’ position, work area, and specialty (*p* < 0.05).

**Table 2:** Patient safety culture dimensions, Fayoum General Hospital, and other selected district hospitals, 2018

Item	Mean percent positive score		Range
	Mean	SD	
Teamwork within hospital units	63.09	12.67	35–100
Staff work conditions	57.6	7.05	40–70
Supervisor/manager expectations and actions promoting safety	59.81	9.83	35–90
Organizational learning-continuous improvement	65.36	15.58	33.3–100
Management support for patient safety	59.5	16.23	20–93.3
Overall perceptions of patient safety	48.34	10.23	16–72
Feedback and communication about errors	20.29	5.01	8.89–28.89
Communication openness	17.96	4.4	8.89–28.89
Frequency of events reported	30.48	10.78	20–80.
Teamwork across units	46.54	10.54	25–85
No. of events	30.48	10.78	20–80
Handoffs and transitions	55.1	16.78	20–9
Non-punitive response to errors	34.74	8.94	20–86.6
Total patient safety score	46.56	3.5	34.8–59.8

More perception of safety dimensions was seen in females than males in dimensions as teamwork within hospital units, management support for patient safety, handoffs and transitions, and non-punitive response to errors. Also, more positive perception of safety dimensions, overall perceptions of patient safety, communication openness, teamwork across units, and number of events reported, was significantly higher in participants with direct contact with patients and in nurses than in technicians and pharmacists. Also, perception of most safety dimensions was higher in participants who had work experience less than 10 years (Table 3).

**Table 3:** Mean scores of patient safety culture dimensions according to demographic characteristics

PSC dimensions	Gender		Direct contact with patients		Position			Length of work	
	Male	Female	Yes	No	Nurse	Technician	Pharmacist	≤ 10 years	> 10 years
Teamwork within hospital units	60.48 ± 12.42	65.59 ± 12.43	63.18 (11.55)	62.81 (15.00)	62.5 ± 11.4	65.1 ± 15.6	62.3 ± 12.6	62.8 ± 12.1	63.4 ± 13.4
Staffing	57.9 ± 6.4	57.3 ± 8.9	57.13 (6.4)	58.7 (8.3)	56.8 ± 6.4	58.9 ± 6.2	59.3 ± 10.4	58.4 ± 7.4	56.6 ± 6.4
Supervisor/manager expectations and actions promoting safety	63.54 ± 9.3	56.16 ± 8.9	59.57 (9.4)	60.38 (10.79)	59.9 ± 9.3	57.7 ± 10.3	62.7 ± 10.9	61.5 ± 9.6	57.6 ± 9.7
Organizational learning—continuous improvement	67.48 ± 17.51	63.28 ± 13.13	63.24 ± 14.35	70.3 ± 17.2	63.8 ± 14.8	67.4 ± 17.4	69.5 ± 15.2	63.5 ± 14.2	67.7 ± 16.9
Management support for patient safety	56.09 ± 12.16	62.89 ± 18.83	58.45 ± 15.49	62.04 ± 17.63	59.2 ± 15.6	57.9 ± 16.6	64.4 ± 17.8	58.3 ± 13.3	61.1 ± 19.3
Overall perceptions of patient safety	47.47 ± 11.13	49.19 ± 9.2	49.28 ± 9.45	46.17 ± 11.59	50.2 ± 9.2	45.5 ± 10.8	44.3 ± 11.9	49.7 ± 9.9	46.6 ± 10.5
Feedback and communication about error	21.38 ± 4.7	19.24 ± 5.1	19.99 ± 4.68	21.01 ± 5.64	20.2 ± 4.7	20.2 ± 5.5	21.1 ± 5.7	20.8 ± 4.6	19.6 ± 5.5
Communication openness	17.5 ± 4.2	18.4 ± 4.5	18.43 ± 4.27	16.89 ± 4.51	18.4 ± 4.3	17.7 ± 4.3	16.3 ± 4.9	17.9 ± 4.3	17.9 ± 4.6
Teamwork across units	46.9 ± 9.9	46.18 ± 11.1	48.01 ± 10.51	43.08 ± 9.78	48.0 ± 10.4	45.3 ± 11.0	41.2 ± 7.8	47.2 ± 10.1	45.7 ± 11.1
Handoffs and transitions	53.6 ± 14.7	56.6 ± 18.5	56.52 ± 15.1	51.80 ± 19.8	57.0 ± 15.1	52.9 ± 17.9	49.3 ± 20.9	52.3 ± 14.5	58.8 ± 18.7
Nonpunitive response to rrrors	33.6 ± 10.5	36.01 ± 6.9	34.86 ± 8.4	34.63 ± 10.19	34.9 ± 8.5	35.0 ± 8.7	33.7 ± 11.4	34.5 ± 9.4	35.2 ± 8.3
Number of event	30.9 ± 11.9	29.9 ± 9.4	31.64 ± 11.4	27.78 ± 8.7	32.0 ± 11.5	27.6 ± 9.2	28.0 ± 7.5	32.2 ± 11.4	28.4 ± 9.5

**Fig. 1:**
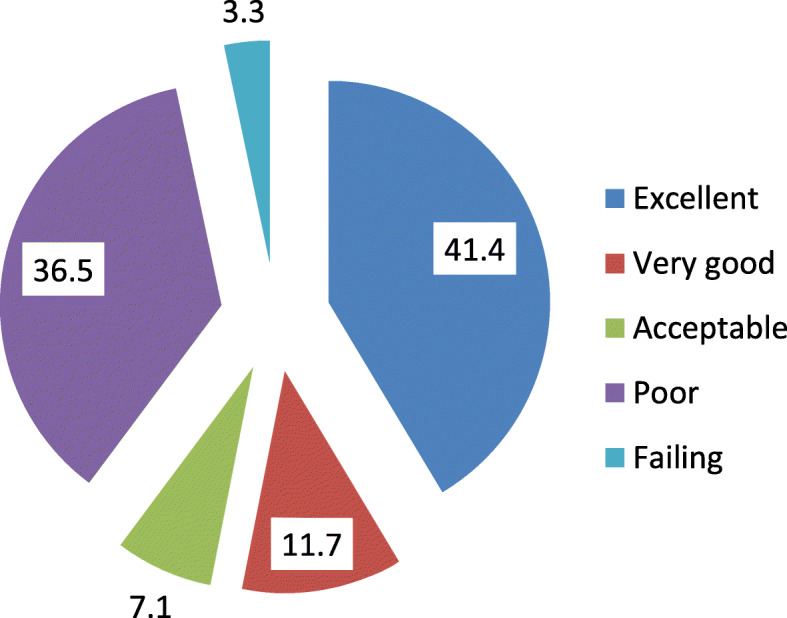
Overall patient safety grade at Fayoum General Hospital and other selected district hospitals, 2018

Participants’ perception of PS grade showed that the proportion of participants who perceived patient safety grade as excellent was 41.3%, while the proportion who perceived patient safety grade as poor was 36.5% and only 3.9% perceived it as failing (Fig. 1).

Table 4 shows the correlations between patient safety culture dimension scores. Nearly each safety culture dimension showed a positive correlation with the other dimensions (*p* < 0.05).

**Table 4:** Correlation between patient safety culture dimension scores

PSC dimensions	Teamwork	Supervision	Organizational learning	Management	Overall	Feedback	Communication	Team unit	Handoff	Punitive	*N* event
Teamwork across hospital units	–	–	–	–	–	–	–	–	–	–	–
Supervisor/manager expectations	0.060	–	–	–	–	–	–	–	–	–	–
Organizational learning	0.220**	0.125**	–	–	–	–	–	–	–	–	–
Management support	0.203**	0.008	0.002	–	–	–	–	–	–	–	–
Overall perceptions of safety	− 0.027	0.186**	0.110*	0.096^*^	–	–	–	–	–	–	–
Feedback about error	− 0.026-	0.328**	0.159**	0.262**	0.138**	–	–	–	–	–	–
Communication openness	0.044	− 0.191**	− 0.149**	0.328**	0.372**	0.144**	–	–	–	–	–
Team unit	0.117*	0.041	0.046	0.006	0.134**	0.081	0.052	–	–	–	–
Handoffs	0.214**	0.174**	0.102*	− 0.070-	0.142**	− 0.036-	0.063	− 0.111*	–	–	–
Punitive	0.108*	− 0.002-	− 0.024	0.143**	− 0.066	0.181**	0.101*	− 0.045-	0.164**	–	–
Number of events	− 0.065	0.011	0.277**	− 0.008	0.239**	0.103*	− 0.018-	0.509**	0.014	0.262**	–
Staffing	− 0.093*	0.140**	0.033	0.094*	0.122**	0.067	− 0.082	− 0.076	− 0.058	0.095*	− 0.073

Pearson correlation, **correlation is highly significant (*p* < 0.0001), *correlation is significant (*p* < 0.001)

## Discussion

Patient safety is a critical component of healthcare quality. Evaluation of safety culture is the primary step towards improving the patient healthcare services in any healthcare organization and investigates the organizational conditions that negatively impact the patient and cause adverse events [13].

The overall mean score for positive perception of patient safety culture dimensions was calculated to be 46.56%, compared to 39.3% by El-Shabrawy et al. [14] in Beni Suef and 69% by Mohamed et al. [15] in Alexandria. In comparison with other Arab countries, these results were lower than the findings documented by Ghobashi et al. in a Kuwaiti study (69%) [16], El-Jardali et al. in Lebanon (61.5%) [17], Alahmadi in Saudi Arabia (61%) [18], and Hamdan and Saleem in Palestine (54%) [19]. Globally, our findings were less than what were detected in China [20], Taiwan [21], the USA [22], and the Netherlands [23] to be 65%, 64%, 65%, and 52.2% respectively, but were higher than a study by Mekonnen et al. (46%) in Ethiopia [24]. These findings were explained by a study done in Egypt [25] as a part of a WHO study in the Eastern Mediterranean region, which addressed the relation between reduced positive perception of patient safety culture dimensions and the culture of blame. The low perception resulted in a decline of the rate of reported errors by 6% which, in turn, contributed to 18% of patient’s permanent disability and mortality rate.

Targeting a positive score above 75% in any domain was reported to be the success level. Unfortunately, in the current study, no domain has achieved this level, and the highest scores were reported for organizational learning-continuous improvement as 65.36% and teamwork within hospital units as 63.09%. The least four dimensions less than 50% that need improvement were non-punitive response to error (34.7%), communication openness (17.9%), feedback and communication about error )20.3% ,(and number of events (30%). These findings were in line with previous findings reported by Aboul-Fotouh et al. [26].

Unlike the findings documented by Mekonnen et al. [24] in Ethiopia with two thirds of staff reported at least one adverse event in the previous year, approximately 44.3% of participants in the current study reported one to two events in the past 1 year. In agreement with the same study, nurses significantly reported better in the overall patient safety score compared with other paramedical personnel. The paramedics of internal medicine were better than the general surgery ones. Additionally, staffing was negatively correlated with all other dimensions except organizational learning and feedback about errors.

The dimension “frequency of adverse events (AEs) reported” had also a low score 30.48%. This could be explained by a lack of a reporting culture and the fact that errors are always considered as a lack of skill not as an opportunity to learn. The staff feels that the errors are alleged against them, and when a mistake is made, they feel that it is the person’s problem. Van Geest and Cummins mentioned three common barriers to report AEs: the punitive systems, humiliation, and fear [27].

The mean composite score for organizational learning-continuous improvement was 65.36% which is less than what was revealed by a study done in a teaching hospital in Egypt to be 78.2% [26], meaning that there is a learning culture only when mistakes are disclosed. A similar finding was reported among Iranian nursing staff (67%) [28] and the hospital staff in Saudi Arabia (75.9%) [29].

In line with other studies [16, 30], the next highest scoring dimension was teamwork within hospital units, 63.09%. This means that people like to actively perform and cooperate with their close peers in the same unit. Similarly, the score of teamwork within units documented in Saudi Arabia in King Fahd general hospital and Ajyad emergency hospital revealed that the teamwork within units for patient safety had 84% positivity [18]. The current study revealed that staffing work condition score was above 50 (57.6%). Duffield et al. **[**31] referred to the association of lack of staff, work overload and unpleasant work environment with the adverse effects on patients, and the occurrence of errors in medical and surgical areas.

The lowest reported score was communication openness (17.9%) which is very low compared with the proportion reported by a study in Kuwait (45%) [16]. That could explain the low number of reported events. A research conducted by Putri et al. addressed that communication openness has a positive and significant effect on the willingness to report patient safety incident [31]. A culture of communication openness in an organization will encourage the feeling of being supported by the managerial office if something is wrong, which will lead to confidence to act appropriately. In other words, the communication needs to be more supportive and open and apply less blame [32]. Handoffs and transition in the current study achieved 55.1% positivity which indicates that there is a real problem regarding the safe continuity of care.

More perception of safety dimensions was seen in females than males in dimensions as teamwork within hospital units, supervisor/manager expectations, and actions promoting safety; this was inconsistent with previous research in Egypt [26] and in Tunisia [33] as they documented no gender difference. The difference may be attributed to the differences in the female to male ratio which was higher in our study (0.98) compared to 0.72 in Tunisia [33] and 0.31 in the Egyptian study [26].

The current study revealed that overall perceptions of PS, communication openness, teamwork across units, handoffs and transitions, and number of events were significantly higher in participants in direct contact with patients which goes along with a study conducted in Kuwait [34].

Participants’ perception of PS grade showed that the proportion of participants’ perceived patient safety grade as excellent was 41.3%. On the other hand, the overall PS was rated as excellent or very good by 60% of respondents in a study done by Alahmadi, in Saudi Arabia [18]. The difference could be the pursuing efforts to improve quality and safety of healthcare services. An initiative has been implemented to improve safety mainly through establishing standards and initiating accreditation schemes in seven developing countries [35].

### Study strengths and limitations

This study was done among healthcare paramedics in secondary-care hospitals belonging to the Ministry of Health in Fayoum Governorate to determine which dimension affects PS culture most. To our knowledge, no study has investigated the perception of patient safety among paramedics. To increase generalizability and strength of the study, this study was carried out in urban and rural health facilities in Fayoum Governorate, with a sufficient sample size and a high response rate of 95.8%. The authors acknowledge some limitations during this study. The first limitation arises from the fact that the data were obtained from paramedics, most of them were nurses, without including physicians. This could be explained by the fact that the main goal of the current study was to focus on this group as was mentioned before. Additionally, the nurses were more available in the hospital during all day and night shifts, so they were the group that most likely to be easily approached for interviews. The study intended to support the concept that paramedic is an essential part of the healthcare team that needs to get attention from stalk holders and be trained on patient safety and identifying the risky behavior and reporting it without fear of repercussions. The second limitation was that the study did not cover the private sector. That was due to the inability of the authors to get the necessary approval.

## Conclusion and recommendations

Overall, the degree of PS is low at Fayoum public hospitals. No dimension had a score above 75%, and seven out of 12 dimensions had scores less than 50%. All patient safety culture dimensions need improvement by continuous monitoring and evaluation to attain a healthy, safe environment for healthcare workers and patients with a great emphasis on communication openness and the number of reported errors.

The paramedics perceived that a “culture of blame” still exists and prevents them from reporting incidents. Thus, managers and supervisors need to educate the healthcare paramedics on the importance of reporting any adverse events and errors to avoid its serious consequences. In addition, creating a web-based incident reporting system accessible for 24 h that allows the healthcare worker to anonymously report the incident using a unique number for each hospital and regularly reviewed by a risk agent for each hospital may encourage reporting of errors.

The Ministry of Health should focus on regularly assessing the safety culture with a standardized assessment tool, providing a basic understanding of the safety-related perceptions of the health staff and evaluating the effectiveness of patient safety programs and interventions. On the educational levels, shifting from traditional training of medical and other clinical skills to train the paramedical students on skills supporting patient safety culture is strongly recommended. Strengthening the integrated health information infrastructure such as electronic medical records to facilitate linking the patient information and capturing the full picture of patient harm is also recommended.

## Data Availability

The dataset supporting the conclusions of this article is available upon request.
